# Stem Cell-Based Therapies in Companion Animals: Molecular Mechanisms, Clinical Applications, and Translational Perspectives

**DOI:** 10.3390/cells15201884

**Published:** 2026-10-15

**Authors:** Weina Liu, Yu Wang, Wei An, Zhenlong Wang, Bing Han, Hui Tao, Yongli Zhang, Fengjiao Gong, Xiumin Wang, Jinquan Wang

**Affiliations:** 1 Key Laboratory of Feed Biotechnology, Ministry of Agriculture and Rural Affairs, Institute of Feed Research, Chinese Academy of Agricultural Sciences, Beijing 100081, China; 2 College of Animal Science and Veterinary Medicine, Shenyang Agricultural University, Shenyang 110866, China; 3 National Nanfan Research Institute (Sanya), Chinese Academy of Agricultural Sciences, Sanya 572024, China

**Keywords:** stem cell therapy, companion animals, regenerative medicine, molecular mechanisms, immunomodulation, translational research

## Abstract

Stem cell-based therapies, as regenerative and immunomodulatory strategies, show substantial potential in companion animals through tissue repair, functional recovery, and immune modulation. Despite advances in human stem cell research, veterinary clinical translation remains constrained by small sample sizes, short follow-up periods, and non-standardized cell dosing regimens and delivery protocols. The expanding repertoire of stem cell-based modalities and technologies—including mesenchymal stem cells, induced pluripotent stem cells, extracellular vesicles, and tissue engineering—provides promising avenues for clinical application. This review summarizes recent progress in stem cell-based therapies across multiple disease systems in dogs and cats, with particular attention to the molecular mechanisms underlying regenerative and immunomodulatory effects, cross-species translational strategies, current challenges, and combinatorial therapeutic approaches in companion animals. By examining mechanistic findings alongside preclinical and early clinical evidence, this review highlights the limitations of current knowledge and identifies research priorities for evaluating the safety and efficacy of stem cell-based therapies in dogs and cats.

## 1. Introduction

Regenerative medicine has reshaped the therapeutic landscape for degenerative diseases, primarily through the distinctive biological properties of stem cells. Stem cells promote tissue repair through two principal pathways: direct differentiation into functional lineages for structural restoration, and paracrine-mediated immunomodulation involving the secretion of growth factors, cytokines, and extracellular vesicles [1,2]. These pathways have been extensively validated in human medicine, particularly with induced pluripotent stem cells (iPSCs) and mesenchymal stem cells (MSCs) in the treatment of osteoarthritis (OA), cardiovascular diseases, and neurodegenerative disorders [3,4,5].

The veterinary field faces an increasingly urgent imperative to integrate stem cell-based therapies into clinical practice. As companion animals live longer, age-related degenerative diseases have emerged as a predominant challenge in veterinary practice. Conventional strategies, ranging from long-term pharmacological management to invasive surgical intervention, are often limited by systemic toxicity, surgical risk, and inadequate regenerative capacity, leaving substantial unmet clinical needs. Concurrently, the global companion animal healthcare market is expanding rapidly—valued at USD 128.9 billion in 2024, it is projected to reach USD 281.54 billion by 2033, underscoring robust demand for advanced therapeutics [6]. These dynamics highlight an urgent need for safer and more effective therapeutic strategies to address these unmet clinical needs.

In recent years, significant advances in human stem cell research—particularly involving iPSCs, MSCs, and genome editing—have demonstrated remarkable regenerative and immunomodulatory potential across cardiovascular diseases, OA, and neurodegenerative disorders [7,8,9]. Given the high degree of conservation in mammalian stem cell biology, these established human-platform technologies are directly relevant to companion animal regenerative medicine. Within the “One Health” framework, convergent progress in human and veterinary science has enabled preliminary validation of key human stem cell attributes in canine and feline models [10]. This cross-species translational potential indicates that adaptation of validated human stem cell protocols holds considerable promise for enhancing therapeutic efficacy and enabling personalized medicine. However, successful translation is not a mere replication of human workflows; it requires rigorous evaluation of interspecies variations in proliferation kinetics, secretome composition, and engraftment efficiency across human, canine, and feline systems.

Despite these advances, evidence from human and veterinary stem cell research remains fragmented across different cell types, disease systems, and species, limiting comparison between studies and translation into veterinary practice. To address this, we conducted a literature review. We searched PubMed and Web of Science for peer-reviewed articles published mainly between 2010–2026, excluding conference abstracts. This review summarizes progress in stem cell-based therapies for dogs and cats, drawing on relevant human studies and experimental animal research. Particular attention is given to distinguishing mechanistic and preclinical findings from clinical evidence in naturally occurring diseases of companion animals. By examining proposed mechanisms, reported therapeutic outcomes, and study limitations, this review aims to identify evidence gaps and priorities for further veterinary research.

## 2. Classification, Characteristics and Mechanisms of Stem Cells

### 2.1. Classification and Characteristics of Stem Cells

Stem cells are conventionally classified into MSCs, iPSCs, neural stem cells (NSCs), etc., according to their tissue origin and differentiation potential, parameters that directly determine their translational prospects (Table 1). Among the various types, MSCs are the most extensively characterized and clinically utilized. MSCs can be isolated from a wide range of tissues, including bone marrow, adipose tissue, umbilical cord blood, placenta, and oral tissues such as dental pulp and periodontal ligament, and they exhibit broad proliferative and multilineage differentiation capacities. In addition, MSCs exert potent immunomodulatory properties primarily through paracrine secretion of anti-inflammatory cytokines and growth factors [2,11]. These properties make MSCs the most mature modality in veterinary medicine, with documented therapeutic efficacy in treating OA, acute hepatic injuries, and cutaneous wounds [12,13,14]. iPSCs, generated by reprogramming somatic cells, possess differentiation potential comparable to embryonic stem cells (ESCs) [15]. They offer a promising avenue for generating patient-specific tissues and are increasingly used for disease modeling, drug discovery, and organoid generation. However, clinical translation of iPSC-based therapies is hindered by unresolved safety issues–particularly incomplete in vivo integration and residual tumorigenicity–which currently limit their veterinary use to ex vivo application. Neural stem cells (NSCs) and ESCs have also been investigated for neurodegenerative disorders and neural repair; nevertheless, ESCs raise ethical concerns and face practical sourcing limitations in companion animals [16,17]. In addition to intact-cell therapies, acellular strategies utilizing stem cell-derived extracellular vesicles (EVs) and decellularized tissue-engineered scaffolds present viable strategies to modulate the regenerative microenvironments. Such approaches can promote tissue repair while circumventing several risks associated with viable-cell transplantation, including immunogenicity, biodistribution control, and long-term survival [18,19].

Notably, the core biological properties of stem cells are highly conserved across mammalian species, facilitating principled cross-species translation of human-derived technologies into veterinary applications. However, interspecies differences in metabolism, immune response, and extracellular matrix composition must be carefully considered when designing clinical regimens, optimizing dose, and evaluating efficacy in target species.

### 2.2. Mechanisms of Stem Cells

**Figure 1:**
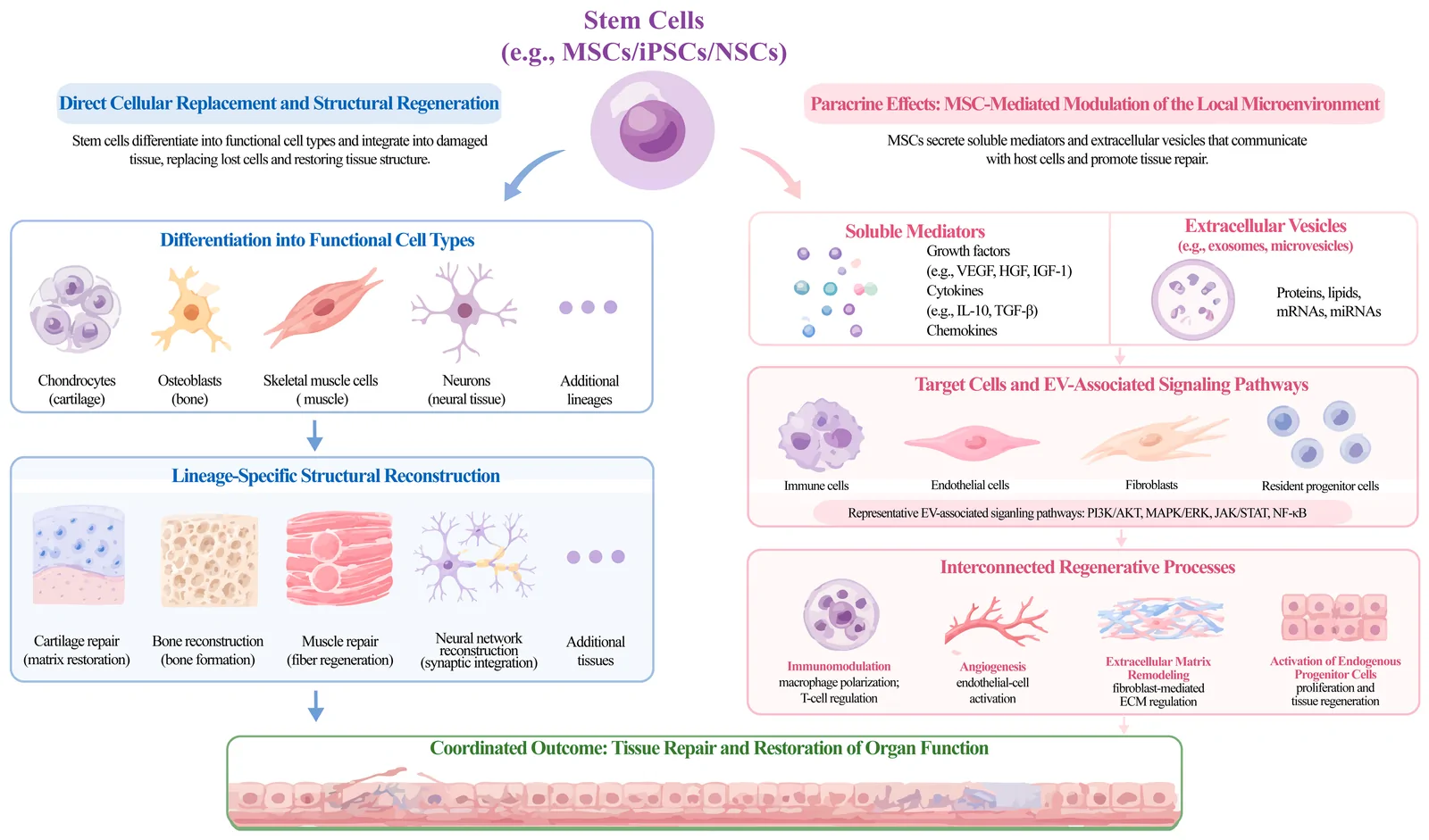
Mechanisms of Stem Cell Action. Notes: VEGF, vascular endothelial growth factor; HGF, hepatocyte growth factor; IGF-1, insulin-like growth factor 1; IL-10, interleukin-10; TGF-β, transforming growth factor beta; PI3K/AKT, phosphoinositide 3-kinase/protein kinase B; MAPK/ERK, mitogen-activated protein kinase/extracellular signal-regulated kinase; JAK/STAT, Janus kinase/signal transducer and activator of transcription; NF-κB, nuclear factor kappa B; ECM, extracellular matrix; mRNAs, messenger RNAs; miRNAs, microRNAs.

Stem cells can contribute to tissue repair and regeneration through direct cellular replacement and paracrine-mediated effects (Figure 1). Direct cellular replacement requires stem cells to differentiate into appropriate cell types and functionally integrate into the damaged tissue. Stem cells can also exert paracrine effects by releasing soluble mediators and extracellular vesicles that modulate host-cell function. These effects have been particularly well characterized in MSC-based studies and can influence multiple host-cell populations through overlapping downstream processes that support tissue repair.

Under the influence of extracellular matrix elasticity, mechanical stimuli, and tissue-specific growth factors, stem cells may differentiate into functional phenotypes such as chondrocytes, osteoblasts, skeletal myocytes, and neurons [35]. Such lineage-specific differentiation enables reconstitution of damaged or depleted cell pools and supports structural reconstruction of injured tissues. For instance, osteogenic differentiation may contribute to bone regeneration [36], whereas neuronal differentiation may facilitate reconstruction of damaged neural networks [37]. Nevertheless, long-term survival, stable engraftment, and durable functional integration of transplanted cells remain limited in many MSC-based studies [38]. The persistence of therapeutic benefits despite limited engraftment suggests that direct cellular replacement alone may not fully account for the observed effects. This has led to increasing attention to paracrine signaling as an important contributor to MSC-mediated tissue repair. However, the relative contribution of direct cellular replacement and paracrine mechanisms may vary across stem cell types and disease models.

MSCs secrete a broad repertoire of bioactive factors, including growth factors, cytokines, and chemokines, together with extracellular vesicles; these components collectively constitute the MSC secretome [38]. These soluble mediators and extracellular vesicles mediate intercellular communication with multiple host-cell populations, including immune cells, endothelial cells, fibroblasts, and resident progenitor cells [39,40]. Extracellular-vesicle cargo can regulate intracellular signaling pathways, including phosphoinositide 3-kinase/protein kinase B (PI3K/AKT), mitogen-activated protein kinase/extracellular signal-regulated kinase (MAPK/ERK), Janus kinase/signal transducer and activator of transcription (JAK/STAT), and nuclear factor kappa B (NF-κB) [41]. Through their actions on these different target-cell populations, MSC-derived paracrine signals contribute to several interconnected regenerative processes, including immunomodulation, angiogenesis, extracellular matrix remodeling, and activation of endogenous progenitor cells. For example, in MSC-based experimental studies, immunomodulatory effects include promotion of macrophage polarization from a pro-inflammatory M1-like phenotype toward an anti-inflammatory and reparative M2-like phenotype and suppression of excessive T-cell activation and proliferation [42,43]. MSC-derived factors can also stimulate endogenous progenitor cells and enhance their proliferation, thereby contributing to tissue regeneration [44]. These processes are neither independent nor strictly sequential; rather, they can share mediators and influence one another within the local tissue microenvironment. Although these mechanisms are supported by experimental evidence, their individual and combined contributions to clinical outcomes in companion animals remain incompletely defined and may vary according to stem cell source, species, disease context, and the local microenvironment. Collectively, stem cell-mediated repair reflects the coordinated contributions of direct cellular replacement, achieved through differentiation and functional integration, and paracrine-mediated effects, with substantial overlap and interaction among the associated regenerative processes.

## 3. Clinical Applications of Stem Cells in Companion Animals

**Figure 2:**
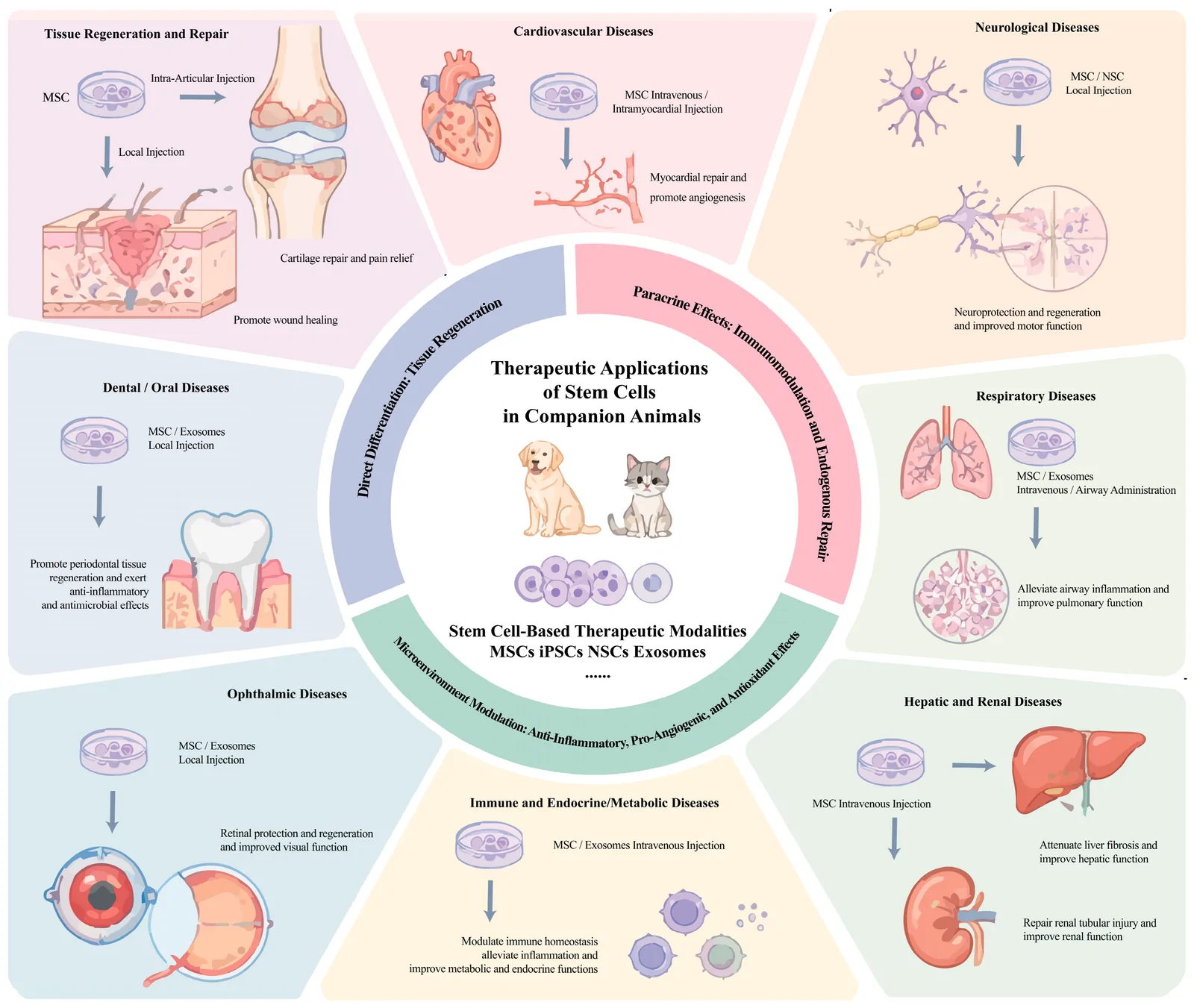
Applications of Stem Cells in Companion Animals.

Stem cell therapies have shown regenerative and immunomodulatory potential across multiple organ systems, although the strength of evidence varies among preclinical studies, experimental canine and feline models, and naturally occurring veterinary diseases (Table 2). These effects may involve direct cellular differentiation together with indirect mechanisms, including immunomodulation, anti-inflammatory activity, paracrine signaling, and local microenvironment remodeling. Among currently investigated platforms, MSCs have accumulated the most extensive veterinary evidence, whereas iPSC-based therapies remain predominantly at the preclinical stage and stem cell-derived extracellular vesicles represent an emerging cell-free approach. As shown in Figure 2, current research focuses not only on optimizing stem cell sourcing and delivery but also on developing combinatorial and adjunctive approaches. Notably, human explorations into multimodal strategies—such as the concurrent use of stem cells with tissue-engineered scaffolds, platelet-rich plasma (PRP), or extracellular vesicles—provide useful conceptual and methodological references for veterinary investigation. Accordingly, adaptation of these strategies requires species-specific validation of cell source, dosing, delivery, and outcome assessment, with standardized and well-controlled studies needed to determine their safety, efficacy, and translational value in dogs and cats.

### 3.1. Osteoarticular and Musculoskeletal Disorders

Osteoarticular and musculoskeletal disorders are common clinical problems in both human and veterinary orthopedics. These conditions include osteoarthritis (OA), articular cartilage lesions, and injuries involving the meniscus, tendons, and ligaments, and are often associated with chronic pain, functional impairment, and reduced quality of life. In human medicine, MSC-based regenerative therapies have been extensively investigated for OA and cartilage repair. Their potential effects include modulation of the intra-articular inflammatory and immune microenvironment through paracrine mediators, including growth factors and cytokines, together with support of chondrogenic repair [45]. Clinical studies have reported improvements in pain and joint function following intra-articular MSC administration, with generally favorable short-term safety findings [45]. In contrast, human induced pluripotent stem cell (hiPSC)-based cartilage regeneration remains predominantly at the preclinical stage. hiPSC-derived mesenchymal progenitors can be directed toward chondrogenic differentiation [101], and specific hiPSC-derived chondroprogenitor populations have shown enhanced expression of cartilage-related markers and increased cartilaginous matrix production [102]. However, differentiation efficiency, cellular heterogeneity, and potential tumorigenic risk remain important barriers to clinical translation. Beyond OA and articular cartilage injury, stem cell-based regenerative strategies are also being investigated for meniscal, tendon, and ligament injuries. Meniscal lesions have limited intrinsic healing capacity because of their relatively poor vascularization, and cell-based therapies and tissue-engineered approaches have therefore been explored as potential regenerative strategies [48]. MSC-based approaches have likewise been investigated for tendon and ligament repair because of their regenerative and immunomodulatory properties [103]. Recent comparative evidence further suggests that cell source may influence tenogenic potential. Tendon-derived MSCs showed stronger tenogenic characteristics than bone marrow-derived MSCs and promoted structural repair in a rat Achilles tendon injury model [49]. In a recent preclinical rat Achilles tendon defect model, adipose-derived MSCs combined with a decellularized tendon scaffold and granulocyte colony-stimulating factor improved collagen organization, tenogenic marker expression, extracellular matrix remodeling, and inflammatory profiles [50]. As this study was conducted in an experimental rat model, its relevance to naturally occurring tendon injuries in companion animals requires further veterinary evaluation. Collectively, these human and preclinical findings provide a biological and translational basis for further evaluation of stem cell-based therapies in veterinary musculoskeletal diseases.

Building on this evidence base, veterinary studies have begun to evaluate stem cell-based therapies directly in companion animals. In companion animals, canine OA is among the better-studied clinical applications of MSC-based therapy, with several controlled or prospective studies contributing to the current veterinary evidence [104]. Autologous adipose-derived stem and regenerative cell therapy improved lameness, pain, and range of motion in dogs with hip OA [12]. In a larger randomized, placebo-controlled trial, xenogeneic equine umbilical cord-derived MSCs improved gait and orthopedic assessment outcomes in dogs with naturally occurring hip or elbow OA; an 18-month owner follow-up further indicated that 59% of respondents perceived a treatment effect lasting longer than 6 months [46]. Additional long-term follow-up of dogs treated with allogeneic adipose-derived MSCs reported no obvious adverse effects over 4–5 years, although sustained clinical improvement was assessed mainly from owner observations [105]. Together, these studies support the therapeutic potential of MSC-based interventions in canine OA and provide preliminary safety data, including long-term follow-up. However, further evidence is needed to establish the consistency and durability of clinical benefits and to more comprehensively evaluate long-term safety in broader canine populations. In an experimentally induced OA model in Beagles, adipose-derived MSCs combined with platelet-rich plasma improved locomotor function and reduced articular cartilage degeneration compared with either treatment alone [47]. However, this study represents preclinical evidence, and its relevance to naturally occurring OA in companion animals requires further validation. Human OA trials may also provide a useful methodological reference for controlled study design and standardized assessment of pain, function, and adverse events in veterinary research [45], while species-specific clinical validation remains necessary for companion animals. Overall, current veterinary clinical studies provide evidence supporting the therapeutic potential of MSC-based treatment for canine OA, whereas applications for meniscal, tendon, and ligament injuries remain more dependent on human and preclinical research and therefore require further species-specific veterinary investigation.

### 3.2. Cardiovascular Diseases

Cardiovascular diseases, including myocardial infarction (MI), cardiomyopathy (CM), heart failure (HF), and valvular heart disease (VHD), represent important clinical challenges in both human and veterinary medicine [106,107]. These conditions can lead to progressive cardiac dysfunction, reduce exercise capacity, impair quality of life, and, in advanced stages, increase morbidity and mortality [108,109]. Stem cell-based regenerative strategies, particularly those involving MSCs and induced pluripotent stem cell-derived cardiomyocytes (iPSC-CMs), have therefore been investigated for cardiac repair and functional recovery. MSCs are thought to exert their effects mainly through paracrine modulation of the myocardial microenvironment, including the release of angiogenic, anti-inflammatory, and immunomodulatory mediators that may support myocardial repair and vascularization [110]. In human studies, the phase I/II POSEIDON trial showed that transendocardial administration of autologous or allogeneic bone marrow-derived MSCs reduced myocardial scar size and favorably affected ventricular remodeling; improvements in functional capacity and quality of life were observed in some groups, whereas overall left ventricular ejection fraction (LVEF) was not significantly increased [111]. A more recent meta-analysis of clinical studies in acute MI reported improvements in LVEF during follow-up of up to 12 months, whereas no significant improvement was observed beyond 12 months and no clear reduction in major adverse cardiac events was demonstrated [112]. Other regenerative approaches remain predominantly preclinical. In a mouse MI model, human umbilical cord-derived MSC sheets improved cell retention, angiogenesis, cardiac function, and ventricular remodeling [113]. Similarly, a meta-analysis of animal studies showed that iPSC-CM transplantation improved cardiac functional parameters and reduced myocardial fibrosis [51]. In addition, the immature structural, metabolic, and electrophysiological phenotype of iPSC-CMs remains an important barrier to clinical translation [114]. Together, these findings indicate that stem cell-based cardiovascular therapies may improve selected measures of cardiac function and remodeling, while their effects on major clinical outcomes, the durability of therapeutic benefit, and the clinical translation of iPSC-CM-based myocardial regeneration remain incompletely defined.

These unresolved questions are equally relevant to companion animals; however, it is important to emphasize that human and preclinical findings do not constitute evidence of established efficacy or long-term safety in veterinary species. Although veterinary clinical evidence remains limited, several studies have explored MSC-based therapies in canine VHD, with findings suggesting potential improvements in cardiac function, clinical status, and disease progression, although the results have not been entirely consistent across studies. In dogs with degenerative VHD, repeated administration of allogeneic puppy deciduous teeth stem cells (pDSCs) was associated with improvements in LVEF, American College of Veterinary Internal Medicine (ACVIM) functional class, and quality of life during a 60-day follow-up [52]. By contrast, a 6-month double-blind, placebo-controlled study of dogs with HF secondary to myxomatous mitral valve disease (MMVD) found that Wharton’s jelly-derived MSCs were well tolerated but did not significantly improve echocardiographic variables, cardiac biomarkers, survival, or diuretic requirements compared with placebo [115]. A more recent study in early-stage MMVD reported relatively stable echocardiographic parameters and slower progression from ACVIM stage B1 to B2 following allogeneic gonadal tissue-derived MSC treatment, although the treated group was small [116]. Thus, current evidence in canine VHD suggests potential clinical benefit, but the magnitude and durability of this benefit remain uncertain. Evidence for other cardiovascular diseases is even more limited. Small studies in dogs with naturally occurring CM have mainly demonstrated treatment feasibility and short-term safety, without consistent evidence of delayed disease progression or improved survival [117,118]. In cats, stem cell treatment has improved selected cardiac functional parameters in an experimentally induced CM model, but the evidence remains preclinical [119]. Overall, evidence for cardiovascular diseases other than VHD is even more limited and largely preliminary: in dogs, studies of naturally occurring CM and other conditions remain small and short-term, while in cats, evidence is still restricted to experimental models.

Human and preclinical cardiovascular studies provide a methodological framework for interpreting veterinary findings; however, these data should not be extrapolated to imply that efficacy or long-term safety has been established in companion animals. In human MSC studies, improvements in LVEF, myocardial scar burden, and ventricular remodeling have been reported, whereas reductions in major clinical events have been less consistent [111,112]. This suggests that veterinary studies should evaluate not only echocardiographic improvement, but also disease progression, functional status, quality of life, survival, and treatment-related adverse effects. In addition, approaches such as MSC sheets, which aim to improve cell retention and local cardiac repair [113], and iPSC-CM transplantation, which targets myocardial replacement but remains limited by cellular immaturity [51,114], may provide directions for future investigation in companion animals rather than representing established veterinary therapies. At present, however, veterinary evidence is still derived mainly from conventional MSC-based studies in dogs, which provide primarily short- to intermediate-term information on treatment effects and tolerability. Whether these observed benefits are sustained over time, and whether long-term safety can be established, remains unclear.

### 3.3. Neurological Disorders and Central Nervous System Injuries

Stem cell-based therapies have been investigated for both neurodegenerative disorders and central nervous system injuries through modulation of the neural microenvironment and, in some cases, cellular replacement. MSCs exert neuroprotective and pro-regenerative effects mainly through the secretion of neurotrophic and immunomodulatory factors that reduce neuroinflammation and support endogenous repair. In neurodegenerative diseases such as Alzheimer’s disease, Parkinson’s disease, and amyotrophic lateral sclerosis, evidence for MSC- and NSC-based approaches remains largely preclinical, with studies suggesting neuroprotective effects and support of neuronal function [120]. Spinal cord injury (SCI), by comparison, has progressed further toward clinical investigation. Experimental studies indicate that MSCs can promote SCI repair through neurotrophic support, immunomodulation, and remodeling of the injury microenvironment [53], while human clinical trials have reported neurological improvement in some patients; however, heterogeneity in cell source, dose, administration route, injury characteristics, and outcome assessment still limits firm conclusions regarding efficacy [54]. iPSC-derived neural cells provide a complementary strategy for neural replacement but remain predominantly preclinical. In rodent SCI models, these cells have demonstrated integration into host tissue, synaptic formation, and axonal extension [121]. Combination strategies involving iPSC-derived motor neural progenitors have further promoted axonal regeneration [122], while iPSC-derived spinal neural progenitors have improved sensorimotor recovery through differentiation and modulation of the injury microenvironment [123]. These findings provide a useful reference framework for evaluating the more limited evidence currently available in companion animals.

In companion animals, research has focused mainly on canine degenerative myelopathy and SCI. In canine degenerative myelopathy, intensive neurorehabilitation combined with allogeneic MSC transplantation was associated with improved functional outcomes and longer survival, although the contribution of MSCs could not be separated from differences in the rehabilitation protocols [55]. For SCI, a pilot study in four dogs with chronic injury found that repeated intravenous administration of allogeneic bone marrow MSC-conditioned medium combined with physiotherapy was well tolerated during 6 months of follow-up and was associated with improved locomotor scores, with bladder function also improving in two dogs [56]. A separate canine case report described improved hindlimb function after decompressive surgery and local administration of amniotic fluid-derived MSCs; magnetic resonance imaging (MRI) showed changes in the lesion signal at 16 weeks that were interpreted by the authors as consistent with axonal remyelination [57]. In dogs with acute thoracolumbar disc-associated SCI, decompressive surgery combined with epidural adipose-derived MSC transplantation shortened locomotor recovery time and hospitalization compared with surgery alone, although the final proportions of dogs recovering locomotion and nociception did not differ significantly [58]. Evidence in cats is much more limited, with a single reported case of chronic compressive SCI showing progressive recovery of pain responses, weight-bearing, locomotion, and partial bowel and bladder function after hemilaminectomy, local autologous bone marrow-derived MSC transplantation, and physiotherapy [124]. Overall, current veterinary studies suggest that stem cell-based approaches may complement surgery and rehabilitation in selected neurological conditions, but their independent therapeutic contribution remains incompletely defined. Human research may provide useful methodological guidance for veterinary studies, particularly regarding standardized motor-function scoring, electrophysiological assessment, MRI, autonomic-function evaluation, and structured rehabilitation protocols.

### 3.4. Organ-Specific Applications

#### 3.4.1. Hepatic and Renal Diseases

MSCs can differentiate into hepatocyte-like cells and modulate the hepatic microenvironment through paracrine secretion of regenerative and immunoregulatory factors, such as hepatocyte growth factor (HGF), epidermal growth factor (EGF), and anti-inflammatory cytokines. MSCs suppress local inflammation and hepatic stellate cell activation while simultaneously promoting hepatocyte proliferation and functional recovery [125,126]. In preclinical models of drug-induced liver injury, cirrhosis, and hepatic fibrosis, MSC transplantation has been shown to improve liver function biomarkers, attenuate fibrosis, and stimulate tissue regeneration [59,127,128]. Furthermore, human iPSC-derived hepatocyte-like cells can acquire hepatic functional characteristics and have been increasingly used for disease modeling and drug discovery, while their regenerative application remains largely preclinical [129,130]. MSC-based therapies have been evaluated in patients with liver cirrhosis and acute liver failure. Meta-analyses have reported improvements in selected liver function parameters without adverse events [131,132]. A long-term randomized study in patients with liver cirrhosis further reported improvements in liver function and long-term survival following umbilical cord-derived MSC treatment [133]. MSCs exert anti-inflammatory, anti-apoptotic, and pro-angiogenic effects that mitigate tubular injury and interstitial fibrosis. Preclinical studies, including evidence from rat acute kidney injury (AKI) models, have shown that MSC transplantation can alleviate renal injury and promote functional recovery [60,134]. A recent review of clinical studies in chronic kidney disease (CKD) indicates that MSC-based interventions have been evaluated for safety, tolerability, and renal functional outcomes, although reported therapeutic responses remain variable [135]. Collectively, these studies may provide a useful reference for future veterinary research, particularly for evaluating liver function and fibrosis progression in hepatic diseases and monitoring long-term changes in renal function in CKD. However, because disease characteristics and therapeutic responses may differ across species, these findings still require validation in dogs and cats and should not be regarded as direct evidence of efficacy for stem cell therapy in companion animals with hepatic and renal diseases.

Renal studies have mainly focused on cats with naturally occurring CKD. A randomized, placebo-controlled trial in eight cats with naturally occurring CKD showed that repeated intravenous MSC administration was well tolerated, but no significant improvement was observed in serum creatinine, blood urea nitrogen, or glomerular filtration rate (GFR) during the 8-week follow-up [61]. A pilot investigation using ultrasound-guided intrarenal MSC injection reported GFR improvements in select CKD cases, suggesting that direct renal delivery needs further investigation [62]. In a canine AKI model, intrarenal administration of canine umbilical cord blood-derived MSCs was associated with reduced blood urea nitrogen and creatinine levels and improved histological findings [63]. A case report describing Fanconi syndrome documented amelioration of renal tubular acidosis and proteinuria following multiple MSC administrations [64]. Compared with renal disease, MSC-based approaches for veterinary hepatic diseases remain at an earlier stage, with current evidence derived mainly from experimental canine hepatic fibrosis models. In these models, bone marrow-derived MSC transplantation significantly reduces fibrotic area and improves liver functional parameters, such as shortened indocyanine green clearance time and decreased fibrosis severity [65,66]. Overall, current veterinary evidence suggests potential biological effects of MSC-based interventions, but their therapeutic efficacy in naturally occurring hepatic and renal diseases remains insufficiently established.

#### 3.4.2. Soft Tissue Injuries and Wound Healing

Cutaneous wound regeneration represents a major focus of tissue engineering and regenerative medicine. Conventional therapeutic strategies often yield suboptimal outcomes in complex or refractory wounds, such as chronic non-healing ulcers and diabetic foot ulcers, providing an opportunity for the clinical application of stem cell therapies. In preclinical and human wound-healing research, MSCs have been investigated primarily for their paracrine and immunomodulatory effects. MSCs secrete bioactive factors, including vascular endothelial growth factor (VEGF), fibroblast growth factor (FGF), and transforming growth factor-β (TGF-β). These paracrine mediators may modulate inflammation, promote angiogenesis and collagen remodeling, and enhance keratinocyte migration [67,136,137]. Experimental studies in acute and chronic wound models support these mechanisms, although their contribution to clinical outcomes may vary according to wound type and treatment approach. Furthermore, iPSC-based approaches remain largely preclinical. In murine wound models, iPSC-derived keratinocytes have been shown to promote re-epithelialization, whereas iPSC-derived endothelial cells enhance neovascularization and wound closure [138,139]. Collectively, these findings provide mechanistic and preclinical support for the potential of stem cell-based approaches to modulate the wound microenvironment, promote angiogenesis, and facilitate tissue repair, although their clinical efficacy in chronic and complex wounds, particularly in companion animals, remains to be established.

Veterinary investigations of stem cell-based wound repair have focused primarily on canine experimental models and a small number of clinical studies. In experimental full-thickness cutaneous wound models in dogs, local MSC administration has been reported to accelerate wound closure and promote collagen deposition and angiogenesis while modulating local inflammatory responses [68]. In a clinical study involving 24 dogs with naturally occurring acute or chronic cutaneous wounds, treatment with allogeneic adipose-derived MSCs was associated with greater wound contraction and re-epithelialization than conventional treatment [14]. Small-scale studies have also evaluated MSC-conditioned media in dogs with complicated non-healing wounds, with favorable wound-area reduction reported following local treatment [69]. In contrast, evidence in cats is much more limited. A recent case report described progressive healing of a chronic non-healing wound following combined treatment with allogeneic adipose-derived MSCs and autologous platelet-rich plasma, although the contribution of MSCs cannot be separated from that of the combined therapy [140]. A recent veterinary review further noted that although MSC-based wound therapies have shown encouraging outcomes across different wound types and treatment protocols, considerable heterogeneity remains among studies [70]. Overall, current evidence has begun to extend beyond experimental wound models to clinical cases, but differences in wound type, treatment protocols, and outcome assessment indicate that the clinical evidence remains preliminary and is not yet sufficient to establish consistent efficacy of MSC-based therapies across naturally occurring wounds in dogs and cats. Human wound research may provide a useful reference for wound-specific outcome assessment, particularly re-epithelialization rate, granulation tissue maturation, and biomechanical strength. These outcome measures still require adaptation and validation in dogs and cats before they can support broader conclusions regarding clinical efficacy.

#### 3.4.3. Respiratory Diseases

MSCs represent a promising regenerative strategy for diverse human pulmonary disorders. Mechanistically, MSCs exert therapeutic effects through immunomodulation, anti-inflammatory responses, and paracrine secretion of trophic factors, which collectively alleviate oxidative stress, reduce inflammatory cell infiltration, inhibit airway remodeling, and ultimately preserve pulmonary structural integrity and function. In preclinical asthma, MSC transplantation significantly reduces eosinophilic inflammation and subepithelial fibrosis while improving histopathological lung injury [141]. Similarly, in emphysema and inflammatory models of chronic obstructive pulmonary disease (COPD), systematic reviews and meta-analyses indicate that MSCs improve alveolar architecture, suppress pro-inflammatory cytokine release, and promote tissue repair [142]. Preclinical studies have shown that in pulmonary fibrosis models, MSC therapy markedly prolongs survival and attenuates fibrosis severity, supporting a disease-modifying potential in chronic interstitial lung disease [143]. Cell-free approaches also remain at the preclinical stage. In rat COPD models, human MSC-derived extracellular vesicles administered to the airways reduced inflammatory responses and improved structural or functional lung outcomes [72,73]. These findings further expand the preclinical evidence supporting MSC-related therapeutic strategies in respiratory diseases and provide a basis for subsequent clinical investigation. Early-phase clinical trials across COPD, interstitial lung disease, and acute respiratory distress syndrome further confirm an acceptable safety profile for MSC products, with favorable trends in inflammatory biomarkers and physiologic indicators such as forced expiratory volume and diffusion capacity, reported in some studies [74,75,76]. These findings provide a useful reference for veterinary evaluation of MSC-based therapies in respiratory diseases, particularly for assessing pulmonary function, inflammatory responses, and airway remodeling, while species-specific validation remains necessary.

Veterinary studies have begun to examine these outcomes in experimental and clinical settings. In feline chronic asthma models, intravenous MSC administration improved airway structural indices and reduced airway remodeling, with concomitant beneficial changes observed on pulmonary imaging; however, the effects on inflammatory cell infiltration and airway hyperresponsiveness were delayed, necessitating further validation [71]. More recently, a case report of a cat with naturally occurring asthma described sustained clinical improvement after intramuscular MSC administration, including discontinuation of corticosteroids and bronchodilators during a 2-year follow-up, although evidence from a single case remains preliminary [144]. Canine evidence has also been reported in an experimental model of radiation-induced lung injury, in which intratracheal transplantation of human umbilical cord-derived MSCs improved arterial oxygenation and attenuated radiographic and histopathological lung injury, accompanied by reductions in inflammatory and fibrotic markers [145]. Cell-free approaches are also being explored in veterinary respiratory research. Canine stem cell-derived extracellular vesicles have shown anti-inflammatory effects in experimental lung-injury systems, including suppression of inflammatory signaling and endothelial-to-mesenchymal transition, although the evidence remains limited to in vitro models rather than naturally occurring canine respiratory disease [146]. Overall, current veterinary studies have reported encouraging findings for MSC-based respiratory interventions, including cell-free derivatives, in experimental models and a small number of clinical cases. However, variability across disease settings highlights the need to determine whether these effects can be reproduced in naturally occurring respiratory diseases in dogs and cats.

#### 3.4.4. Immune-Mediated and Inflammatory Diseases

Stem cell-based therapies have emerged as a promising regenerative strategy aimed at modulating immune responses and facilitating endogenous tissue repair. MSCs can regulate systemic immune homeostasis through paracrine secretion of anti-inflammatory cytokines, such as TGF-β and interleukin-10 (IL-10), and through direct intercellular interactions that promote regulatory T-cell responses and suppress effector lymphocyte activation [147]. These immunomodulatory effects have been further demonstrated in preclinical models of autoimmune and inflammatory diseases. In experimental systemic lupus erythematosus (SLE), MSC-mediated immunomodulation was associated with reduced T helper 17 (Th17) responses, decreased interleukin-17A (IL-17A) levels, increased interleukin-4 (IL-4) expression, and attenuation of inflammatory injury in affected tissues [77]. Similarly, in murine models of inflammatory bowel disease (IBD), MSCs and MSC-derived acellular products alleviated intestinal inflammation by reducing lymphocyte infiltration and pro-inflammatory signaling, while improving mucosal injury and barrier-related changes [148,149]. These experimental findings are complemented by growing human clinical evidence. A systematic review and meta-analysis of randomized controlled trials across autoimmune and rheumatic diseases reported improvements in disease activity in several conditions, including SLE and IBD, without an overall increase in adverse events [78]. Likewise, early clinical studies in rheumatoid arthritis (RA) demonstrated that MSC therapy improved arthritis activity scores and reduced inflammation markers while exhibiting a favorable safety profile [79]. Additionally, MSC-derived extracellular vesicles and engineered cell-sheet constructs have been investigated as emerging strategies to enhance immunomodulation and accelerate tissue regeneration in animal preclinical settings [150,151], suggesting potential complementary approaches for immune-mediated diseases. Collectively, MSC-based approaches have accumulated substantial preclinical and early clinical evidence supporting their utility in human immune-related diseases. Compared with conventional treatments that mainly suppress immune activation, MSC-based strategies may offer the additional advantage of simultaneously modulating dysregulated immune responses and promoting endogenous tissue repair. Despite these potential advantages, clinical responses remain variable across different immune-mediated inflammatory diseases, and these findings require further validation before they can be extrapolated to companion animals.

Against this broader background of MSC-mediated immunomodulation, veterinary clinical studies have begun to evaluate MSC-based interventions in naturally occurring immune-mediated inflammatory diseases in dogs. In canine IBD, a single infusion of allogeneic MSCs, administered either alone or in combination with prednisone, was associated with sustained improvements in clinical disease activity, serum albumin, and cobalamin during 12 months of follow-up, while corticosteroid dosage was progressively reduced in dogs receiving combined treatment [80]. In canine immune-mediated hemolytic anemia, intravenous administration of allogeneic MSCs as an adjunct to standard immunosuppressive therapy resulted in hematologic improvement, including an increase in packed cell volume, while no serious infusion-related adverse events were reported [81]. Similarly, repeated administration of canine adipose-derived MSCs in a dog with refractory immune-mediated thrombocytopenia (IMT) was followed by disease remission, withdrawal of prednisolone, and improvement of complications associated with prolonged immunosuppressive treatment [82]. However, the evidence for IMT is currently limited to a single clinical case. These preliminary data suggest that MSCs have potential to modulate aberrant immune responses and improve clinical outcomes in companion animals. Drawing upon the methodological framework established in human MSC studies, future veterinary investigations should integrate comprehensive assessments encompassing hematologic indices, immune activity, and histopathological evaluation-to better define therapeutic efficacy and safety. Because several veterinary studies involve concurrent immunosuppressive treatment or are based on limited clinical cases, the independent contribution of MSCs remains to be clarified. Emerging approaches, including MSC-derived extracellular vesicles, also warrant further evaluation in veterinary immune-mediated inflammatory diseases.

#### 3.4.5. Endocrine and Metabolic Diseases

MSCs, iPSCs, and their derivatives, particularly insulin-producing islet-like cells, have been investigated for their regenerative and metabolic effects in diabetes, pancreatic β-cell dysfunction, and related metabolic disorders. Studies utilizing diabetic mouse models have shown that MSC transplantation can reverse hyperglycemia and improve glucose tolerance. Experimental evidence suggests that MSC-secreted growth factors (e.g., insulin-like growth factor, IGF) and anti-inflammatory molecules may contribute to reducing islet cell apoptosis and enhancing β-cell survival and function [152]. Concurrently, in vitro and in vivo investigations confirm that iPSCs can differentiate into functional islet-like cells capable of glucose-responsive insulin secretion, providing a theoretical basis for β-cell replacement—a core strategy in cellular therapies for diabetes [84]. Human clinical evidence has also begun to emerge. In patients with type 1 diabetes, allogeneic umbilical cord-derived MSC transplantation resulted in improvements in β-cell functional outcomes, including C-peptide-related measures, together with better glycemic control, while no severe treatment-related adverse events were reported [85]. Furthermore, stem cell-derived exosomes have demonstrated immunomodulatory, anti-inflammatory, and β-cell-protective effects in preclinical diabetes studies [86], providing a potential cell-free strategy for regenerative therapy. Collectively, these studies support the potential of MSCs and iPSC-derived islet-like cells in diabetes treatment. However, clinical evidence for MSC-based approaches remains limited, while iPSC-derived islet-like cells are still largely at the preclinical stage. This distinction is important when considering their therapeutic role, as stem cell-based approaches are being explored for regenerative effects beyond the glycemic control achieved with conventional insulin and glucose-lowering therapies. For iPSC-derived islet-like cells, differentiation efficiency, tumorigenic risk, and long-term functional stability remain important considerations. Human studies may nevertheless provide a methodological reference for veterinary assessment of glycemic control, endogenous insulin secretion, and insulin requirements, while therapeutic efficacy requires independent validation in dogs and cats.

Veterinary clinical evidence is currently represented mainly by a small number of canine studies. A pilot study involving four dogs with insulin-dependent diabetes mellitus (IDDM) reported that intravenous administration of allogeneic adipose-derived MSCs improved appetite, body weight, and activity levels. Furthermore, improvements in C-peptide, glycated hemoglobin A1c, and fructosamine suggested a possible enhancement of endogenous insulin secretion and metabolic control [83]. Beyond direct MSC administration, canine studies have also explored β-cell replacement strategies using adipose-derived MSCs. In one study, canine adipose-derived MSCs were reprogrammed into islet β-like cells and cotransplanted with MSCs in both experimentally induced diabetic dogs and two clinical canine diabetes cases. The treatment produced favorable metabolic responses, supporting the feasibility of this approach for canine diabetes, although the clinical evidence remains limited [153]. Other preclinical studies have shown that canine adipose-derived MSCs can be differentiated into glucose-responsive insulin-producing cells, although transplantation has so far been evaluated mainly in experimental systems rather than naturally occurring canine diabetes [154]. Similar work has also been reported in cats. In vitro studies have shown that feline adipose-derived stromal cells can be differentiated into glucose-responsive insulin-producing cell clusters, providing a species-specific cellular basis for further preclinical evaluation of β-cell replacement strategies in feline diabetes [155]. However, these approaches have not yet been clinically established for diabetes in dogs or cats. Overall, current veterinary evidence suggests potential metabolic benefits of MSC-based interventions in canine diabetes, particularly in glycemic control and β-cell-related function, while direct clinical evidence in companion animals remains limited. Future veterinary studies should focus on disease-specific outcomes, including insulin requirements, C-peptide, fructosamine, and clinical signs, and clarify whether the effects observed with emerging preclinical strategies can be reproduced in naturally occurring diabetes.

### 3.5. Special Senses and Oral Cavity

#### 3.5.1. Ophthalmic Diseases

MSCs, retinal stem cells (RSCs), and iPSC-derived retinal cells have been increasingly investigated for ocular tissue repair. Preclinical studies have shown that MSCs can promote corneal repair. In a rat model of corneal scarring, MSC-derived exosomes significantly reduced inflammatory cytokine levels, accelerated epithelial closure, and promoted wound healing, indicating that MSC paracrine factors play a crucial role in corneal repair [156]. In a rat model of retinal degeneration, combined transplantation of human iPSC-derived retinal pigment epithelial (RPE) cells and retinal precursor cells (RPCs) preserved the outer nuclear layer and visual responses, while transplanted RPE cells showed integration into the host RPE layer [157]. Recent reviews have also summarized early clinical investigations of MSC-based therapies for retinal degenerative diseases, with preliminary improvements in visual and structural outcomes reported in some studies, although their clinical efficacy remains under evaluation [158]. Furthermore, tissue-engineered scaffolds designed for the delivery of RSCs and retinal progenitor cells have been explored in preclinical retinal models to improve transplanted-cell survival, distribution, and integration within the subretinal space, thereby enhancing overall transplantation efficacy [159]. These findings highlight the promise of stem cell-based therapies for promoting corneal repair and retinal protection. Rather than replacing conventional ophthalmic treatments, these regenerative strategies may provide complementary options for tissue repair and cell replacement, particularly in conditions involving irreversible corneal scarring or photoreceptor loss. However, most of the current evidence remains preclinical, and whether these regenerative effects can translate into consistent clinical benefits and be reproduced in naturally occurring ocular diseases in companion animals remains to be determined.

Direct veterinary clinical evidence is currently focased mainly on corneal disease. In dogs with naturally occurring deep corneal ulcers that had failed to heal after previous medical treatment, local administration of allogeneic MSCs resulted in complete ulcer healing within 14 days in 22 of 26 treated dogs, accompanied by reduced ulcer depth and alleviated ocular discomfort [88]. Similarly, in feline corneal ulceration and perforation cases, MSC-treated animals achieved more rapid restoration of corneal integrity and earlier fluorescein-negative conversion compared with medical treatment alone [89]. Recent reviews have also noted consistent trends toward reduced inflammation and improved tissue repair indicators in companion animals with ocular surface diseases, supporting a rationale for more systematic investigations [70]. Together, these findings suggest that MSC-based approaches may facilitate corneal healing in dogs and cats, although the available studies differ in design and treatment protocols. To better characterize therapeutic responses, veterinary studies may adopt outcome measures commonly used in human ophthalmic research, distinguishing early healing indicators such as epithelial closure and fluorescein-negative conversion from longer-term structural and functional outcomes, including corneal transparency and visual function, with appropriate adaptation to canine and feline models.

#### 3.5.2. Dental and Oral Diseases

Stem cell therapies for dental and oral regeneration have increasingly focused on MSC populations derived from craniofacial tissues, including dental pulp, exfoliated deciduous teeth, periodontal ligament, and bone marrow. These cells possess osteogenic, cementogenic, and odontogenic differentiation potential that is relevant to alveolar bone, periodontal tissue, and dentin–pulp regeneration. Among them, dental pulp stem cells (DPSCs) and periodontal ligament stem cells (PDLSCs) have attracted particular attention because of their proliferative capacity and multilineage differentiation potential. DPSCs are closely associated with dentin–pulp regeneration, whereas PDLSCs are particularly relevant to the reconstruction of periodontal ligament, cementum, and alveolar bone. Preclinical evidence further supports the regenerative potential of both cell populations. A network meta-analysis of periodontal regeneration studies showed that DPSCs and PDLSCs promoted the formation of new alveolar bone, cementum, and periodontal ligament, providing experimental support for their use in periodontal tissue reconstruction [160]. Human clinical studies have also begun to evaluate these oral tissue-derived stem cells directly. In a 12-month prospective pilot trial, autologous PDLSCs combined with a xenogeneic bone substitute improved clinical attachment level and probing pocket depth in patients with periodontal intrabony defects, although the additional benefit over the bone substitute alone did not reach statistical significance [91]. More recently, a multicenter randomized clinical trial involving 132 patients with chronic periodontitis evaluated allogeneic DPSCs and reported greater improvement in bone defect depth than saline treatment, with additional improvement in attachment loss observed in patients with stage III periodontitis [161]. A systematic review and meta-analysis further reported improvements in clinical attachment level, probing depth, and periodontal bone-defect parameters following stem cell-based therapy [93]. In parallel, tissue-engineering approaches have combined stem cells with scaffold materials to provide a three-dimensional microenvironment that supports cell adhesion, proliferation, and differentiation, thereby promoting coordinated regeneration of periodontal soft and hard tissues [94]. Together, these findings demonstrate the regenerative potential of oral tissue-derived stem cells and related stem cell-based strategies in human and preclinical dental research and provide a relevant basis for examining whether comparable effects can be achieved in companion animals.

Direct canine studies have begun to address this question at both the cellular and tissue levels. Canine DPSCs can be isolated from dental pulp and display typical MSC characteristics, including plastic adherence, multilineage differentiation, appropriate surface-marker expression, and proliferative and colony-forming capacity, supporting dental pulp as an alternative source of MSCs for canine regenerative applications [25]. More recently, canine DPSCs exposed in vitro to biomaterials relevant to regenerative endodontics maintained viability and odontogenic/osteogenic differentiation potential, providing further evidence for their compatibility with cell–material approaches to pulp regeneration [95]. In canine periodontal defects, transplantation of PDLSC-based tissue-engineering scaffolds promoted new alveolar bone and periodontal ligament formation, while reducing local pro-inflammatory responses [26]. Direct in vivo evidence has also been obtained for canine pulp regeneration. In pulpectomized dog teeth, transplantation of hypoxia-preconditioned DPSCs generated regenerated pulp tissue comparable in quantity and quality to that obtained with mobilized DPSCs, without evidence of transplantation-related toxicity or adverse events [96]. In aged dogs, in which pulp regenerative capacity is reduced, transplantation of mobilized DPSCs together with a C-C chemokine receptor type 3 (CCR3) antagonist increased regenerated pulp tissue and was accompanied by neovascularization and reinnervation, indicating that the age-related local microenvironment can influence the regenerative response [97]. These observations are consistent with evidence that the dental pulp microenvironment regulates the homeostasis, proliferation, differentiation, and regenerative activity of odontogenic MSCs [98]. Cell-free derivatives of oral tissue-derived stem cells are also being explored. Canine DPSC-derived exosomes have recently been characterized as containing proteins related to inflammation regulation, angiogenesis, extracellular matrix organization, cell migration, and tissue remodeling [162]. Although their initial xenogeneic application was evaluated in feline cutaneous wound healing rather than oral disease, the study provides preliminary evidence for the feasibility of canine DPSC-derived extracellular vesicles as a veterinary cell-free regenerative platform. Together with small extracellular vesicles derived from dental follicle stem cells [99], these findings extend oral stem cell research beyond direct cell transplantation toward cell-free regenerative strategies. Human dental regenerative research may provide useful methodological references for veterinary studies, particularly through imaging modalities, periodontal probing, quantitative alveolar bone assessment, pulp vitality, dentin formation, vascularization, and innervation.

## 4. Stem Cell Therapeutic Strategies and Cross-Species Translational Insights

### 4.1. Technologies and Therapeutic Applications

In the clinical development of stem cell therapies for companion animals, the optimization of both technological platforms and therapeutic strategies is critical for improving treatment consistency and safety. The choice of cell sources and types can influence therapeutic performance. MSCs derived from bone marrow, adipose tissue, and umbilical cord are among the most extensively investigated cell types in dogs and cats, although their biological properties may vary according to tissue sources, donor characteristics, and culture conditions. In addition, cell expansion, cryopreservation, and other manufacturing procedures can affect cell quality and functional activity, highlighting the need for standardized production, quality-control, and potency-testing procedures for veterinary MSC products [163,164]. iPSCs and their derivatives represent a promising alternative for tissue regeneration, but their veterinary application remains largely preclinical. In addition, stem cell-derived extracellular vesicles or microvesicles offer a cell-free therapeutic avenue that modulates immune responses and restores tissue homeostasis through paracrine mechanisms. Their lower immunogenicity, small size, and amenability to storage and engineered delivery further support their therapeutic potential in veterinary regenerative medicine [165,166].

Apart from cell selection, the optimization of delivery routes and dosing regimens is also important for the clinical translation of stem cell-based therapies. Commonly adopted administration methods in veterinary studies include intravenous injection, local injection, intra-articular delivery, and scaffold- or carrier-assisted implantation. The selection of delivery routes can impact cell retention, biodistribution, and exposure to the target tissue. For instance, intra-articular MSC injection has been investigated for OA in both human and canine studies [47,167], whereas systemic intravenous delivery is associated with broader biodistribution and distinct cell-homing patterns [168]. Notably, in addition to optimizing delivery, more attention has been given to combinatorial and adjunctive strategies, including MSCs combined with PRP, biomaterial scaffolds, other stem cell populations, or conventional treatments. Representative strategies and their primary outcomes across different disease systems are summarized in Table 3. Several comparative studies have shown that combination therapies can provide additional benefits in cell retention, tissue repair, and functional recovery compared with single treatments, highlighting their potential to enhance therapeutic effects through complementary mechanisms. However, the strength and nature of the supporting evidence vary among different combinations and study designs. Therefore, their therapeutic advantages should be interpreted within the context of the specific model, species, and evidence level.

Standardized evaluation frameworks are essential for assessing the reproducibility and clinical relevance of stem cell therapies. In veterinary clinical studies, efficacy is commonly evaluated using disease-specific imaging modalities (e.g., MRI, X-ray, joint ultrasonography), functional assessments (e.g., gait analysis, cardiac function scoring), and biochemical parameters (e.g., hepatic, renal, and inflammatory biomarkers). Safety should be assessed independently through adverse-event monitoring and long-term follow-up. In experimental studies, these evaluations may be further complemented by histological analysis, cell tracking, and assessment of cellular engraftment and tissue repair. Greater harmonization of study endpoints, follow-up intervals, and long-term monitoring would facilitate comparison across studies and improve the quality and reproducibility of veterinary evidence. Overall, standardized cell preparation, delivery strategies, dosing regimens, and outcome assessment are important for supporting the evidence-based clinical translation of stem cell therapies in companion animals. When applied to combinatorial approaches, such standardized frameworks are particularly needed to distinguish demonstrated synergy from promising experimental combinations across heterogeneous models.

### 4.2. Cross-Species Translation of Stem Cell Therapies

**Figure 3:**
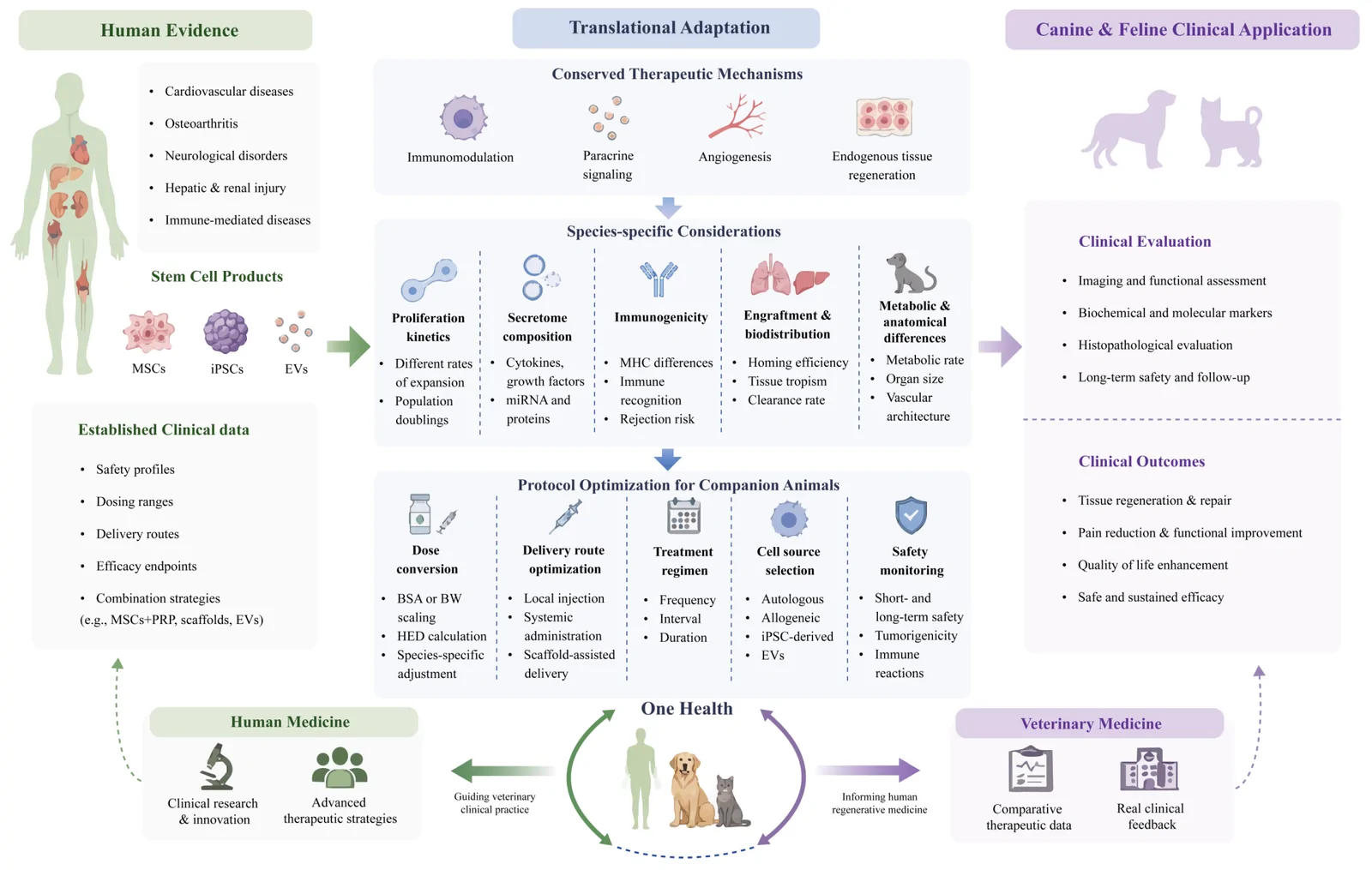
Translational Framework of Stem Cell Therapies from Human Evidence to Canine and Feline Clinical Applications. Notes: MHC, major histocompatibility complex; BSA, body surface area; BW, body weight; HED, human equivalent dose.

Stem cell therapies in companion animals can adopt human clinical and preclinical experience. Under the “One Health” concept, conserved biological properties of stem cells across mammalian species provide a basis for human-derived data to guide experimental design and therapeutic strategies in dogs and cats. Human stem cell research has generated substantial methodological experience in safety assessment, dosing, administration routes, and efficacy evaluation, particularly for MSC-based therapies, whereas iPSC-based strategies remain at earlier and more variable stages of clinical translation. Additionally, combinatorial strategies developed in human medicine, such as the combined use of stem cells with tissue-engineered scaffolds, PRP, or extracellular vesicles, provide conceptual frameworks for veterinary investigation. Nevertheless, direct translation of human protocols requires careful consideration of species-specific biological differences. Canine and feline stem cells have been reported to differ from their human counterparts in proliferation capacity, secretory profiles, engraftment behavior, and immunomodulatory activity, which may influence therapeutic responses [163,164]. Therefore, dosing regimens, delivery routes, and efficacy evaluation strategies in humans may serve as methodological starting points, but must be adapted to the physiological and pathological characteristics of the target species and validated in species-specific studies before any clinical recommendations can be made (Figure 3).

In practice, cross-species dose selection for stem cell therapies should consider body weight, metabolic characteristics, disease site, administration route, and stem cell biodistribution rather than relying on a simple dose-conversion formula. Current veterinary clinical studies show substantial heterogeneity in cell source, preparation, dose, administration route, and outcome measures, which limits direct comparison between studies and complicates the establishment of standardized treatment protocols [104]. Accordingly, reported doses should be interpreted as study-specific examples rather than generalized recommendations. For instance, one clinical study of canine OA reported weight-adjusted intra-articular doses of 4–30 × 10^6^ autologous adipose-derived MSCs per joint in combination with PRP [173]; however, this dosing range reflects a single study design and should not be interpreted as an established clinical guideline. Delivery route is another important consideration that should be selected according to disease type and target tissue accessibility. Local administration, including intra-articular, cutaneous, or myocardial delivery, has been used when direct access to the affected tissue is feasible, whereas intravenous administration enables systemic delivery but may be limited by pulmonary first-pass effects and subsequent cell redistribution [168]. Scaffold- or carrier-assisted delivery has also been explored to support local cell retention and persistence, while combinations with PRP or rehabilitation have been investigated as adjunctive therapeutic approaches (Table 3). Cross-species protocols therefore require careful consideration of dose, delivery route, local retention, and functional persistence of transplanted cells. These parameters should be adapted and validated according to the target species, disease characteristics, and available veterinary evidence, rather than directly extrapolated from human protocols.

Beyond these species- and treatment-specific considerations, the translational readiness of different stem cell-based platforms varies considerably. Given the distinct development stages of MSCs, iPSCs, and stem cell-derived extracellular vesicles, each therapeutic platform should be evaluated according to its own evidence base rather than assumed to have equivalent clinical applicability across species. Relevant human clinical experience can provide methodological guidance, but species-specific validation remains necessary. Future veterinary studies should prioritize systematic comparisons of cell sources and therapeutic platforms in dogs and cats, using human clinical experience to guide the evaluation of delivery methods, combinatorial strategies, and outcome assessment. Such efforts may support a more consistent and evidence-based pathway for clinical translation; however, current evidence does not support generalized dosing recommendations, and any cross-species translation of human dose ranges should be presented as illustrative examples rather than clinical guidelines.

## 5. Challenges and Future Perspectives

Despite promising findings from preclinical studies and selected veterinary clinical reports, the application of stem cell therapy for multi-system diseases in companion animals currently faces several key challenges. First, existing studies are frequently constrained by small sample sizes and short follow-up periods, resulting in insufficient evidence regarding the durability of therapeutic effects and long-term safety. This limitation restricts robust assessment of clinical efficacy and hinders the establishment of standardized, generalizable treatment protocols. Second, substantial heterogeneity exists regarding stem cell types (MSCs, iPSCs, and their derivatives), tissue sources (bone marrow, adipose tissue, umbilical cord), donor characteristics, cell expansion and cryopreservation procedures, dosing regimens, and administration routes. In addition, differences in manufacturing standards, quality control, and potency testing may further affect product consistency and complicate comparisons across studies. Such variability makes it difficult to identify reproducible dosing strategies, treatment frequencies, and therapeutic responses. Third, safety concerns differ among therapeutic platforms. Tumorigenic risk remains particularly relevant to iPSC-based products, whereas immune reactions and other long-term transplantation-related effects require continued evaluation for cell-based therapies. Safety and efficacy should therefore be assessed as distinct outcomes, supported by standardized adverse-event monitoring and disease-specific efficacy endpoints. The absence of unified clinical assessment metrics and post-treatment monitoring systems further complicates regulatory evaluation and broader clinical adoption. Finally, species-specific biological differences limit the direct translation of human-derived protocols, emphasizing the need for species-specific validation in canine and feline species before broader clinical implementation.

To overcome these barriers, future research should prioritize large-scale, multicenter, randomized controlled trials to establish standardized criteria for stem cell sourcing, administration protocols, and evaluation frameworks, thereby generating more robust and long-term clinical evidence. Greater attention should also be given to standardizing cell manufacturing, quality control, potency assessment, and long-term safety monitoring to improve the reproducibility of veterinary stem cell products and clinical studies. Additionally, combinatorial strategies integrating tissue-engineered scaffolds, stem cell-derived extracellular vesicles, platelet-rich plasma, or rehabilitation warrant further evaluation as approaches to improve local delivery, cell retention, and complementary therapeutic effects. Within the “One Health” framework, relevant human clinical experience, combined with species-specific validation, may help refine cross-species applications and reduce translational uncertainty. Emerging approaches that integrate longitudinal clinical monitoring, computational analysis, and experimental validation may also support more individualized optimization of treatment protocols, although their utility in veterinary stem cell therapy remains to be established. Overall, current evidence warrants further investigation of stem cell-based therapies in selected disease areas in veterinary medicine, but claims of broad therapeutic potential across multiple disease categories are premature. Broader veterinary application will depend on generating stronger, standardized, and long-term clinical evidence.

## 6. Conclusions

Stem cell therapies have demonstrated biological plausibility as a regenerative and immunomodulatory strategy for multisystem disorders in companion animals, with promising findings reported across musculoskeletal, cardiovascular, neurological, hepatic, renal, and immune-related disorders. However, these promising findings derive largely from preclinical models or small, heterogeneous veterinary clinical studies, and clinically established efficacy has not yet been robustly demonstrated in most indications. Existing studies are frequently constrained by small sample sizes, short follow-up durations, heterogeneity in cell sources and treatment protocols, and unresolved interspecies biological differences. Accordingly, cautious interpretation is required, and promising preclinical findings should not be equated with clinically validated therapeutic efficacy. Rather than representing standardized or reliably established treatment options, current stem cell applications in veterinary medicine should be viewed as evolving investigational approaches. This review has summarized current advances, mechanistic evidence, and emerging combinatorial strategies, while highlighting the urgent need for standardized evaluation frameworks. Future research may focus on comparative evaluation of stem cell sources, refinement of delivery technologies, and the implementation of sufficiently powered, long-term clinical studies to independently establish therapeutic efficacy and long-term safety. Only through such rigorous evidence generation can standardized, evidence-based regenerative therapies for companion animals be realistically contemplated. Broad clinical guidelines and reliable therapeutic applications remain long-term objectives that are contingent upon overcoming the current methodological and evidentiary limitations.

## Figures and Tables

**Table 1:** Categories and Properties of Stem Cells.

Stem Cell Type	Main Source	Differentiation Potential	Key Characteristics	Major Applications/Limitations	References
Embryonic Stem Cells (ESCs)	Inner cell mass of early embryos	Pluripotent	Differentiation into all three germ layers; high proliferative capacity	Ethical controversies; high tumorigenicity; potential immune rejection	[20,21]
Adult Stem Cells (ASCs)	Adult tissues (bone marrow, adipose tissue, nervous tissue, etc.)	Multipotent/Unipotent	Wide availability; low immunogenicity; minimal ethical concerns	Limited differentiation potential; lower ex vivo expansion capacity	[22,23]
Mesenchymal Stem Cells (MSCs)	Bone marrow, adipose tissue, umbilical cord, placenta, dental pulp, periodontal ligament, etc.	Multipotent	Strong immunomodulatory capacity; differentiation into bone, cartilage, and adipose tissue	High heterogeneity; characteristics vary by tissue source	[11,24,25,26]
Hematopoietic Stem Cells (HSCs)	Bone marrow, umbilical cord blood, peripheral blood	Multipotent	Differentiation into all blood cell lineages; essential role in hematopoietic system reconstruction	HLA matching requirement; risk of graft-versus-host disease	[27,28]
Neural Stem Cells (NSCs)	Embryonic and adult brain tissue, spinal cord	Multipotent	Differentiation into neurons, astrocytes, and oligodendrocytes	Limited availability; difficult ex vivo expansion; low directed differentiation efficiency	[16,29]
Induced Pluripotent Stem Cells (iPSCs)	Somatic cells (reprogrammed)	Pluripotent	ESC-like pluripotency; absence of ethical controversy; patient-specific customization potential	Tumorigenic risk (residual reprogramming genes); need for further safety optimization	[15,30]
Perinatal Stem Cells	Umbilical cord blood, amniotic fluid, placenta	Multipotent/Lineage-restricted	Abundant source; low immunogenicity; high proliferative capacity	Insufficient long-term safety data; lack of standardized isolation and storage protocols	[31]
Germline Stem Cells (GSCs)	Testes, ovaries	Multipotent/Unipotent	Differentiation into gametes; maintenance of fertility	Difficult isolation and in vitro culture; application largely restricted to reproductive medicine	[32,33]
Stem Cell Derivatives (EVs)	Secreted products from various stem cells	None (signal delivery)	Paracrine effects; absence of cell-therapy safety risks; modulation of immune response and microenvironment	Non-unified purification standards; long-term efficacy and stability requiring further validation	[34]

Notes: HLA, human leukocyte antigen; EVs, extracellular vesicles.

**Table 2:** Comparative Overview of Stem Cell-Based Therapies in Humans and Companion Animals.

Disease Types	Cell Types	Application Comparison		Efficacy	Safety/Limitations	Strategies	References
		Human	Companion Animals				
Osteoarticular/Musculoskeletal	MSCs/iPSC -derived chondrogenic cells	MSCs clinically studied for OA; iPSC cartilage strategies mainly preclinical	Canine OA; experimental MSC + PRP; limited tendon/ligament evidence	Pain and gait improvement; cartilage repair	Evidence strongest for OA; tendon/ligament data mainly preclinical	Standardized gait/imaging; long-term studies	[45,46,47,48,49,50]
Cardiovascular	MSCs/pDSCs/iPSC-CMs	MSC clinical/preclinical studies; iPSC-CMs mainly preclinical	Limited canine studies	Improved selected cardiac indices and clinical scores	Small samples; short follow-up	Optimizing cell source, route, and cardiac monitoring	[51,52]
Neurological/Spinal Cord Injury	MSCs/NSCs/iPSC-derived neural cells	Broad preclinical evidence; limited clinical translation	Experimental and naturally occurring canine SCI	Motor and functional improvement	Small, heterogeneous studies; concurrent treatments	Standardized motor scoring, MRI, long-term follow-up	[53,54,55,56,57,58]
Hepatic/Renal	MSCs/iPSC-derived hepatocyte-like cells	Early liver clinical evidence; renal evidence mainly preclinical	Feline CKD; canine AKI/Fanconi syndrome; experimental canine liver disease	Renal responses inconsistent; hepatic improvement mainly experimental	Small cohorts; limited clinical evidence	Organ-specific outcomes and long-term controlled studies	[59,60,61,62,63,64,65,66]
Cutaneous Injuries	MSCs/MSC-conditioned medium	Human and preclinical wound studies	Experimental and naturally occurring canine wounds; limited feline evidence	Wound closure and reepithelialization	Heterogeneous wounds and treatments	Standardized wound-healing endpoints	[67,68,69,70]
Respiratory	MSCs/MSC-derived EVs	Early human studies; EVs mainly preclinical	Feline asthma and canine lung injury models	Reduced airway remodeling; inflammatory responses inconsistent	Sparse, mainly experimental evidence	Imaging, cytology, pulmonary function	[71,72,73,74,75,76]
Immune-Mediated/Inflammatory	MSCs/EVs	Human clinical/preclinical evidence	Canine IMHA, IMT, and IBD	Improved selected hematologic/clinical indices	Small studies; concurrent immunosuppression	Integrated immune and long-term safety assessment	[77,78,79,80,81,82]
Endocrine/Metabolic	MSCs/iPSC-derived β-like cells	Human and preclinical diabetes studies	Limited canine studies; feline evidence mainly in vitro	Changes in glycemic indices	Very limited veterinary clinical evidence	HbA1c, fructosamine, C-peptide, long-term follow-up	[83,84,85,86,87]
Ophthalmic	MSCs/iPSC-derived retinal cells	Mainly preclinical corneal and retinal studies	Naturally occurring canine/feline corneal disease	Corneal healing and fluorescein-negative conversion	Small/retrospective studies; limited retinal evidence	Corneal transparency and visual-function follow-up	[88,89]
Dental/Oral	DPSCs/PDLSCs	Human/preclinical periodontal and pulp regeneration	Canine periodontal and pulp regeneration	Periodontal and pulp tissue regeneration	Mostly experimental canine evidence	Imaging, periodontal and pulp-specific outcomes	[25,26,90,91,92,93,94,95,96,97,98,99,100]

Notes: OA, osteoarthritis; PRP, platelet-rich plasma; pDSCs, puppy deciduous teeth stem cells; iPSC-CMs, induced pluripotent stem cell-derived cardiomyocytes; SCI, spinal cord injury; MRI, magnetic resonance imaging; CKD, chronic kidney disease; AKI, acute kidney injury; IMHA, immune-mediated hemolytic anemia; IMT, immune-mediated thrombocytopenia; IBD, inflammatory bowel disease; HbA1c, glycated hemoglobin A1c; DPSCs, dental pulp stem cells; PDLSCs, periodontal ligament stem cells.

**Table 3:** Representative Combinatorial and Adjunctive Stem Cell-Based Strategies Across Disease Systems.

Disease System	Combinatorial or Adjunctive Strategy	Species/Model	Evidence Level	Reported Outcomes	References
Osteoarticular	AD-MSCs + PRP	Beagle dogs, induced OA	Canine comparative study	Improved lameness, cartilage properties, and histology versus single treatments	[47]
Osteoarticular	BM-MSCs + PRF scaffold	Dogs, osteochondral defects	Canine preclinical study	Enhanced cartilage repair	[169]
Osteoarticular	MSC exosomes + icariin hydrogel	Rats, OA	Rodent comparative study	Reduced cartilage degeneration and improved exosome retention	[170]
Cardiovascular	iPSC-CMs + MSCs	Mice, myocardial injury	Rodent comparative study	Improved cardiac function and reduced scar area	[171]
Neurological	iPSC motor neuron progenitors + engineered MSCs	Rats, chronic SCI	Rodent comparative study	Enhanced functional recovery and axonal regeneration	[123]
Neurological	Decompression + canine AD-MSCs	Dogs, thoracolumbar SCI	Veterinary comparative study	Shortened locomotor recovery and hospitalization	[58]
Dental/oral	Canine PDLSCs + scaffold	Dogs, periodontal defects	Canine preclinical study	Promoted alveolar bone and periodontal regeneration	[26]
Hepatic	AD-MSCs + silk fibroin scaffold	Mice, liver fibrosis	Rodent preclinical study	Reduced fibrosis and liver injury	[172]
Cutaneous	Canine amniotic MSC-conditioned medium + standard care	Dogs, non-healing wounds	Veterinary comparative study	Greater wound-area reduction	[69]
Immune-mediated	Allogeneic MSCs + prednisone	Dogs, naturally occurring IBD	Veterinary clinical study	Improved disease activity and reduced corticosteroid use	[80]

Notes: AD-MSCs, adipose-derived mesenchymal stem cells; BM-MSCs, bone marrow-derived mesenchymal stem cells; PRF, platelet-rich fibrin.

## Data Availability

No new data were created or analyzed in this study. Data sharing is not applicable to this article.
